# Seed Amplification Assay for α-Synuclein: Diagnostic Applications in Synucleinopathies

**DOI:** 10.3390/ijms26167817

**Published:** 2025-08-13

**Authors:** Alexandros Giannakis, Louisa Pechlivani, Chrissa Sioka, George Alexiou, Spiridon Konitsiotis, Athanassios P. Kyritsis

**Affiliations:** 1 Department of Neurology, Faculty of Medicine, School of Health Sciences, University of Ioannina, University Campus, 45500 Ioannina, Greece; papadates@gmail.com (A.G.); skonitso@uoi.gr (S.K.); 2 Neurosurgical Institute, University of Ioannina, 45500 Ioannina, Greece; louisapechlivani@gmail.com (L.P.); csioka@yahoo.com (C.S.); galexiou@uoi.gr (G.A.); 3 Department of Nuclear Medicine, Faculty of Medicine, School of Health Sciences, University of Ioannina, University Campus, 45500 Ioannina, Greece; 4 Department of Neurosurgery, Faculty of Medicine, School of Health Sciences, University of Ioannina, University Campus, 45500 Ioannina, Greece

**Keywords:** seed amplification assay, α-synuclein, Parkinson’s disease, dementia with Lewy bodies, multiple system atrophy, diagnosis

## Abstract

Seed amplification assays (SAA) targeting misfolded α-synuclein have emerged as powerful tools for the diagnosis and study of synucleinopathies, including Parkinson’s disease (PD), dementia with Lewy bodies, and multipßle system atrophy. These assays exploit the prion-like seeding properties of pathological α-synuclein to detect minute amounts of misfolded protein in biological specimens. the PubMed database was searched according to our study criteria, and 55 clinical studies comprised the final literature review. the majority of studies have focused on patients at various stages of PD, with cerebrospinal fluid (CSF) being the most commonly investigated biological specimen. Diagnostic utility was most pronounced in the CSF of PD patients, whereas results from other biological samples and across different synucleinopathies have been more modest. α-syn SAA demonstrate significant diagnostic potential in synucleinopathies. Additional applications may include monitoring disease progression. Future studies should explore the utility of α-syn SAA in alternative biological specimens, assess its performance across various synucleinopathies and other neurodegenerative diseases, and determine its comparative diagnostic value.

## 1. Introduction

Parkinson’s disease (PD) is the second most common neurodegenerative disorder, following Alzheimer’s disease (AD) [1]. Although patients with PD often respond well to antiparkinsonian medications in the early and moderate stages of the disease, long-term treatment is inevitably accompanied by adverse side effects that significantly diminish quality of life [2]. Moreover, none of the currently approved therapies have been shown to meaningfully alter the course of disease progression [3].

The situation is even worse for dementia with Lewy bodies (DLB), a syndrome that shares many clinical features with PD, including parkinsonism, idiopathic rapid eye movement sleep behavior disorder (iRBD), dysautonomia and cognitive decline, but the response to levodopa is generally poor, resulting in greater disability and worse prognosis [4,5,6]. Common clinical findings often result in misdiagnosis between these two syndromes [7]. Similarly, multiple system atrophy (MSA), another neurodegenerative disease, in which dysautonomia is more pronounced and other clinical features, such as ataxia may also be mistaken for PD [7,8]. Apart from stress to the patient and patient’s relatives, misdiagnosis may result in unnecessary interventions, and wrong enrollment of patients in studies that help us understand the pathogenesis of these diseases and/or, hopefully, lead us to a disease-modifying treatment [9].

Several biomarkers, mostly imaging, have been proposed as supportive or exclusive for the diagnosis of PD, DLB, and MSA, including dopamine transporter single-photon emission computed tomography (DAT-SPECT) uptake as exclusive criterion for PD, the cingulate island sign in the fluorodeoxyglucose—positron emission tomography (FDG-PET) as supportive for DLB, and the “hot-cross bun” sign in the magnetic resonance imaging (MRI) for MSA [4,5,8]. However, these biomarkers reflect structural and/or functional alterations, in contrast to other neurodegenerative proteinopathies such as AD, where biomarkers directly target the underlying biological pathology, including amyloid beta and tau proteins [10].

The shared pathological hallmark of PD and DLB is the presence of Lewy bodies—neuronal cytoplasmic inclusions composed of pathologically misfolded alpha-synuclein (α-syn), a presynaptic protein with various cellular functions [11]. Like PD and DLB, multiple system atrophy (MSA) is characterized by pathological cytoplasmic inclusions of misfolded α-syn. However, in contrast to the other two synucleinopathies, α-syn aggregation in MSA primarily occurs in both glial cells, particularly oligodendrocytes, and neurons [12]. Like AD, a shift toward a biological definition of PD, centered on α-syn, has recently been proposed [13]. Beyond enabling more accurate disease-specific diagnoses, this approach could also facilitate the development of targeted therapies [3]. Therefore, establishing reliable diagnostic biomarkers that can accurately detect α-syn and distinguish PD from other synucleinopathies, such as DLB and MSA, is of paramount importance.

More than two decades have passed since Braak et al. proposed their groundbreaking hypothesis of α-syn pathology propagating through the brain in a characteristic spatiotemporal pattern, placing this process at the center of pathogenesis in both PD and DLB [14]. In fact, these processes appear to take place several years before the clinical syndrome emerges [15]. The prion-like behavior of misfolded α-syn, where pathogenic species seed the misfolding of native α-syn molecules, has drawn parallels with the self-replicating mechanism of the pathological scrapie prion protein in Creutzfeldt–Jakob disease [16,17]. This mechanistic similarity has prompted researchers to adapt ultrasensitive detection methods originally developed for prion diseases, including real-time quaking-induced conversion (RT-QuIC) and protein misfolding cyclic amplification (PMCA) [18,19]. These techniques, collectively termed seed amplification assay (SAA), exploit the self-templating capacity of misfolded proteins to amplify minute amounts of pathological seeds [20,21]. In recent years, SAA has emerged as a promising diagnostic biomarker for PD, and related synucleinopathies, targeting their pathogenetic core, and demonstrating high sensitivity and specificity in cerebrospinal fluid (CSF) and other biospecimens [21]. However, several challenges have emerged regarding the potential clinical application of SAA [21].

This narrative review aims to summarize key findings from studies using SAA to detect α-syn in human specimens from individuals with PD, DLB, MSA, or those at risk of developing a synucleinopathy.

## 2. Results

### 2.1. Populations at Risk of Developing a Synucleinopathy

As previously mentioned, α-syn aggregates can appear in the central nervous system several years before the onset of clinical symptoms associated with synucleinopathies [15]. Consequently, in the context of early intervention —and to avoid unnecessary treatments that may lead to serious side effects such as psychosis or impulse control disorders [22]—numerous studies have investigated the accuracy of the SAA in detecting α-syn in populations at risk of developing such disorders. Kluge et al. employed blood samples from individuals with and without risk factors for PD to identify pathological α-syn conformers derived from neuronal extracellular vesicles using SAA [23]. Remarkably, all patients who eventually developed PD within a follow-up period of 4 to 10 years had tested positive for α-syn SAA—not only at the point of clinical diagnosis but also as early as 1 to 10 years prior [23]. In contrast, all healthy controls who did not develop PD consistently tested negative for α-syn SAA. Furthermore, approximately 30% of patients with iRBD, also tested positive for α-syn [23].

iRBD is known prodromal condition for synucleinopathies, including PD, DLB, and MSA [24]. Other studies have also investigated α-syn SAA in iRBD. Concha-Marambio et al. have used SAA to detect α-syn in the CSF of newly diagnosed PD patients, healthy controls, and individuals with iRBD [24]. After 10 years of longitudinal follow-up, including standardized clinical evaluation and DAT-SPECT, they found an even greater SAA CSF positivity for the iRBD group, compared to Kluge et al. (93%), while 98% of PD patients were tested positive [24,25]. Interestingly, SAA showed greater agreement with the final diagnosis, which in 14 cases changed to a non-α-syn proteinopathy. Additionally, SAA showed higher diagnostic efficacy compared to DAT-SPECT [24].

On the other hand, when Brown et al. investigated α-syn SAA positivity in the CSF of individuals over 60 years old with hyposmia—another prodromal feature of PD—and abnormal DAT-SPECT findings, the results were more modest [26]. α-syn SAA positivity ranged from 55% to 70%, depending on the severity of hyposmia [26]. Similarly, Yan et al. conducted a retrospective study involving individuals with various risk factors for developing PD, including iRBD, hyposmia (with available DAT-SPECT imaging), carriers of mutations in the beta-glucocerebrosidase (*GBA1*) gene—known to be associated with PD—sporadic PD patients, and healthy controls [27]. Using SAA to assess α-syn positivity in the CSF, they found that positive results were primarily observed in individuals with a greater than 80% probability of prodromal PD [27]. Individuals with prodromal exhibit non-motor symptoms associated with the disease, such as iRBD, dysautonomia and hyposmia, indicating a high-risk for phenoconversion to typical PD, but had not yet developed motor manifestations [27].

In a similar manner, Siderowf et al. evaluated α-syn SAA positivity in the CSF of individuals with iRBD, hyposmia, and carriers of *GBA1* or leucine-rich repeat kinase 2 (*LRRK2*) gene mutations (with or without PD), alongside healthy controls [28]. The assay effectively distinguished patients with sporadic PD from healthy controls, demonstrating particularly high diagnostic accuracy in PD patients with typical hyposmia (98.6% positivity) [28]. However, α-syn SAA positivity rates were notably lower—ranging from 34.7% to 78.3%—among individuals with *LRRK2* mutations and/or normal olfaction [28].

Hence, genes associated with PD also appear to influence α-syn SAA positivity in various specimens, even among at-risk populations [29]. Interestingly, another study reported that 27.8% of patients with Gaucher’s disease type 1—caused by mutations in the *GBA1* gene—tested positive for α-syn SAA in skin biopsies, despite only one of them having clinically established PD [29].

Notably, individuals with a positive α-syn SAA appear to be at increased risk not only for PD but also for other synucleinopathies. In a recent study, Mastrangelo et al. repeatedly performed α-syn SAA on CSF samples from patients with PD, including those who were cognitively unimpaired, had mild cognitive impairment, or had dementia [30]. The researchers conducted the assay using four replicates per sample, assessing both the number of positive replicates and the time-to-threshold (TTT)—the duration required for the fluorescence signal to reach the threshold. They found that individuals who initially tested negative but later converted to positive had longer lag times and fewer positive replicates in follow-up assessments, compared to those who tested positive from the outset [30]. Interestingly, TTT was negatively associated with the progression of dementia in both the overall cohort and the PD subgroup. In contrast, the number of positive replicates at baseline strongly predicted the development of dementia in both groups. The authors concluded that progression of α-syn pathology in patients with Lewy body disease, which includes both PD and DLB—is reflected in dynamic changes in α-syn SAA parameters [30].

Similarly, Coughlin et al. used several amplification parameters, such as maximum fluorescence, time to reach the fluorescence threshold, and half-time to reach maximum fluorescence, in individuals with PD, prodromal PD, non-symptomatic carriers of gene mutations associated with PD, such as *GBA1*, *LRRK2*, and *SNCA* (gene coding α-syn), and healthy controls [31]. Similarly to Mastrangelo et al., individuals with prodromal PD and positive α-syn SAA had higher rates of phenoconversion to clinically established PD [30,31]. Moreover, several amplification parameters, including TTT, maximum fluorescence (Fmax), time-to-50% Fmax (T50), and fluorescence area under the curve (AUC) differed significantly in the prodromal and established PD groups compared to mutation carriers and healthy controls [31]. However, half of the 48 patients with prodromal PD did not develop PD, despite longitudinal follow-up [32].

Palmqvist et al., using participants from the same cohort, found that only individuals who tested positive for α-syn SAA at baseline progressed to Lewy body disease—either PD or DLB [33]. Furthermore, α-syn SAA positivity was associated with a more rapid decline in cognitive function, particularly in attention/executive functioning and memory [33]. Subsequently, the same study group found that patients who were α-syn SAA positive exhibited faster cognitive decline, regardless of whether they had a baseline diagnosis of PD or DLB, or the presence of co-pathologies, 48% of whom also had AD pathology [34].

Similarly, Goldstein et al. assessed α-syn SAA positivity in individuals presenting with three or more risk factors for developing Lewy body disease, such as iRBD, dysautonomia, or genetic mutations, and followed them over a 7.5-year period [35]. They found that 64% of individuals who tested positive at baseline eventually developed Lewy body disease, compared to only 5% of those who initially tested negative [35].

Lastly, 48% of individuals with hyposmia had positive α-syn SAA in their CSF, compared to those with normal olfaction. However, only 35% of these individuals developed dopaminergic deficits on DAT-SPECT over the four-year follow-up period [36].

### 2.2. Parkinson’s Disease

#### 2.2.1. Cerebrospinal Fluid

CSF is the biological fluid in which α-syn SAA has been most extensively investigated in PD. Table 1 summarizes the main findings of studies that investigated α-syn SAA in the CSF of individuals with PD.

Shahnawaz et al. initially recruited patients with PD and compared them to patients with AD, other neurodegenerative diseases, and other neurological conditions [37]. Their objective was to assess whether protein misfolding cyclic amplification’s ability to detect extremely small amounts of misfolded α-syn oligomers, by nucleating aggregation and amplifying the fluorescent signal, could identify PD patients and correlate with disease progression [37]. They found that CSF α-syn SAA positivity could detect PD with both sensitivity and specificity [37]. Additionally, they reported a correlation between SAA kinetic parameters, namely T50, and symptom severity as measured by the Hoehn and Yahr scale (H&Y) [37]. Bhumkar et al. subsequently examined a small sample of patients with PD compared to healthy controls [38]. Six of eight PD patients had positive CSF for α-syn SAA, compared to one of eight controls [38].

Larger studies that compared PD patients to normal controls followed. Orrù et al. investigated α-syn SAA results in the CSF of patients with moderate to advanced PD, comparing them to healthy controls [39]. The assay was positive in 97% of PD patients, versus only 13% of healthy controls, yielding an AUC of 0.95 [39]. Other amplification parameters—Fmax, TTT, T50, and fluorescence AUC—did not show statistically significant group differences, apart from a negative correlation between T50 and scores on the iRBD questionnaire [39].

To examine the method’s reproducibility, Russo et al. performed α-syn SAA in three different laboratories, utilizing the CSF of patients with either early or de novo PD (contrarily to Orrù et al.), healthy controls, and patients with parkinsonism but no evidence of dopaminergic deficits in DAT-SPECT [39,40]. They repeated the test after a three-year follow-up. Notably, they exhibited concordant results among the three laboratories, with high sensitivity and specificity [40]. Interestingly, positive samples from patients with either inconsistent clinical diagnosis or normal DAT-SPECT later progressed to clinically established PD [40]. The study of Oftedal et al. also included recently diagnosed PD patients (α-syn SAA of their CSF maximum 38 days after the diagnosis) but found slightly lower sensitivity and specificity [41].

Notably, Majbour et al. utilized a α-syn oligomer-specific (Enzyme-linked Immunosorbent Assay) ELISA, in addition to α-syn SAA, to examine α-syn SAA in the CSF of PD patients and healthy controls [42]. ELISA-added SAA achieved complete sensitivity and specificity for PD patients [42]. Moreover, α-syn oligomer levels in CSF were positively correlated with motor disability scores [42]. Middleton et al. utilized DAT-SPECT in addition to CSF α-syn SAA to evaluate the latter’s diagnostic accuracy among PD patients and healthy controls [43]. α-syn SAA demonstrated moderate diagnostic accuracy in clinically established PD, which improved when combined with DAT-SPECT [43].

Several CSF elements may influence seed quantification. To investigate this, Bellomo et al. employed a wide range of techniques, including CSF fractionation, mass spectrometry, immunoassays, and transmission electron microscopy, to examine α-syn oligomer interactions with other CSF components in patients with PD, normal pressure hydrocephalus, and healthy controls [44]. Interestingly, they found that CSF apolipoproteins, specifically ApoA1 and ApoE, strongly inhibited α-syn aggregation [44]. They also observed α-syn–apolipoprotein complexes and reported correlations between ApoA1 and ApoE levels and SAA kinetic parameters [44]. Another possible implication is the presence of co-pathology. Gaetani et al. recruited patients with PD, AD, or other neurological diseases and examined the co-occurrence of the two proteinopathies and their correlation with markers of synaptic dysfunction, namely synaptosomal-associated protein 25 kDa (SNAP25) and vesicle-associated membrane protein 2 (VAMP2), in CSF and plasma [45]. They found that in AD patients with α-syn SAA positivity, SNAP25 levels in serum and CSF, and VAMP2 levels in CSF, remained stable across all AD stages [45]. In contrast, PD patients positive for AD-related biomarkers showed higher SNAP25 levels in serum and plasma compared to those negative for AD biomarkers, suggesting that these synaptic markers are primarily influenced by AD- rather than PD-related pathology [45].

CSF α-syn SAA has also been used to evaluate drug response. Eijsvogel et al. applied CSF α-syn SAA in PD patients treated with UB-312, an α-syn–targeting immunotherapy [46]. A significant reduction in CSF α-syn seeds was observed in the treatment group, indicating potential utility as a PD biomarker for target engagement [46].

As already mentioned in the populations-at-risk subsection, genetic mutations associated with PD may impact α-syn SAA results. Grillo et al. recruited 176 genetic PD patients (with *LRRK2* or *GBA1* mutations) and 371 sporadic PD patients [47]. *LRRK2* mutation carriers exhibited lower CSF α-syn SAA positivity and specificity compared to *GBA1* carriers and sporadic PD patients [47]. Kinetic parameters also differed significantly between *LRRK2* carriers and the other study groups [47]. Notably, within the sporadic PD group, the subgroup with dysautonomia showed shorter T50 and larger fluorescent AUC, regardless of the presence of RBD [47].

Lastly, Bräuer et al. examined α-syn SAA kinetic parameters and the detergent resistance of distinct α-syn aggregates in PD patients and healthy controls [48]. Additionally, they transfused the amplified fibrils into cultured cells and assessed α-syn pathology using staining for phosphorylated α-syn, followed by automated high-throughput microscopy [48]. Two types of aggregates appeared to exist, distinguished by their kinetic profiles and cell seeding capacities, potentially contributing to the clinical heterogeneity observed in PD [48].

#### 2.2.2. Other Biological Specimens

Due to the invasive nature of CSF collection, α-syn SAA has been evaluated in a variety of other types of biological samples. Table 2 summarizes the main findings of these studies.

Blood is an attractive and minimally invasive biofluid for detecting α-syn, however, several challenges compromise the reliability and reproducibility of this approach. The vast majority of α-syn present in blood originates from peripheral sources—primarily red blood cells and platelets—rather than the central nervous system (CNS) [58,59,60]. As a result, hemolysis can lead to significant contamination, and rigorous purification protocols are essential [58,59]. Additional contamination may arise from abundant plasma proteins such as albumin [58]. To address these limitations, many studies have focused on isolating neuronal exosomes (NEs), which circulate in the bloodstream but originate in the CNS [58]. These vesicles offer a promising strategy for selectively measuring α-syn that is truly CNS-derived, thereby improving both the specificity and reliability of detection [58].

Schaeffer et al. utilized NEs to detect α-syn in the blood of patients with PD and healthy controls over a longitudinal follow-up period of 5 to 9 years [49]. The assay demonstrated high sensitivity in distinguishing PD cases [49]. Notably, patients with longer disease duration exhibited reduced α-syn seeding activity, as reflected by lower fluorescence intensity at the endpoint of the amplification assay (F60) [49]. These findings suggest that seeding activity in NEs may serve not only as a diagnostic marker but also as a potential biomarker for disease progression [49].

Blood α-syn SAA has also been employed to investigate genetic forms of PD. Kluge et al. evaluated α-syn SAA in NEs from blood in relation to pathogenic *PRKN* mutations, comparing PD patients with *PRKN* mutations, sporadic PD cases, and healthy controls [25]. They found that 8 of 13 *PRKN* mutation carriers were positive for α-syn SAA, suggesting that *PRKN* may be linked to CNS α-syn pathology [25]. Additionally, Daida et al. investigated the *V15A* variant of *SNCA* in patients with familial PD, sporadic PD, and HCs [50]. *V15A*-derived α-syn fibrils exhibited greater amplification activity in the SAA compared to wild-type α-syn, suggesting enhanced seeding potential associated with this rare variant [50].

Wang et al. applied α-syn SAA to both serum and saliva samples from PD patients and HCs [51]. Serum αSyn-SAA demonstrated moderate sensitivity but high specificity and accuracy, while saliva αSyn-SAA showed modest sensitivity with slightly higher specificity and comparable accuracy [51]. Notably, the combined use of serum and saliva αSyn-SAA achieved the highest sensitivity, specificity, and overall accuracy [51]. Additionally, serum seeding activity correlated negatively with Montreal Cognitive Assessment (MoCA) scores and positively with Hamilton Depression Rating Scale scores in females and patients below 70 years [51]. In contrast, they found a negative correlation between saliva α-syn SAA and age at diagnosis in males and in patients below 70 years of age [51].

Saliva has also been studied as an alternative to CSF for α-syn SAA. Given that α-syn is nearly ubiquitous in the body and pathologic α-syn is thought to propagate in a rostro-caudal manner—potentially originating in the gastrointestinal tract—the salivary glands are considered part of the early rostral regions along this pathway [52]. Chahine et al. compared α-syn SAA in CSF and saliva from PD patients and HCs [52]. While CSF samples demonstrated high sensitivity and specificity, saliva yielded more modest results [52]. Notably, only 65.8% of patients were positive for α-syn SAA in both CSF and saliva [52].

Given that the gastrointestinal tract is considered one of the initial sites of pathological α-syn aggregation, with subsequent propagation to the CNS, studies have extended α-syn SAA testing to GI-derived specimens beyond saliva. Vascellari et al. applied α-syn SAA to duodenal biopsies from PD patients and healthy controls [53]. Remarkably, the assay demonstrated exceptionally high diagnostic sensitivity and specificity [53]. However, no significant correlation was found between α-syn seeding activity and clinical parameters such as disease duration, Movement Disorders Society Unified Parkinson’s Disease Rating Scale Part III (MDS-UPDRS III) scores, or constipation severity, suggesting limited utility for monitoring disease progression [53]. Similarly, Fenyi et al. conducted α-syn SAA on gastrointestinal biopsies obtained from the antrum, sigmoid colon, and rectum in PD patients and healthy controls [54]. However, while the assay demonstrated exceptionally high specificity, sensitivity remained low [54]. Notably, most positive cases were detected in biopsies from the sigmoid colon [54]. On the other hand, Emmi et al. combined gastric, duodenal, and skin biopsies to evaluate α-syn SAA, recognizing that pathological α-syn aggregates can also be detected in peripheral tissues such as skin [55]. They tested samples from PD patients with varying disease durations and from healthy controls. Diagnostic accuracy was highest for skin biopsies, outperforming both gastric and duodenal samples [55]. However, gastric biopsies demonstrated superior specificity compared to the other regions in patients with advanced PD [55]. Similarly, Shin et al. applied α-syn SAA to biopsies from the upper and lower gastrointestinal tract of PD patients and healthy controls, but only 10% of gastric biopsies from PD patients tested positive [56].

Lastly, skin biopsy has also been examined alongside brain autopsy tissue in a study by Mao et al., who employed a modified seed amplification assay known as quiescent SAA [57]. This approach was designed to avoid the repeated fragmentation seen in standard SAA, which can interfere with tracking α-syn propagation [57]. Using specimens from patients with PD, individuals without synucleinopathies, and healthy controls, quiescent SAA successfully amplified α-syn aggregates from brain tissue [57]. Moreover, it demonstrated high sensitivity and specificity in skin biopsies for distinguishing PD patients from both non-synucleinopathy cases and healthy controls [57].

### 2.3. Dementia with Lewy Bodies

Apart from PD, α-syn SAA has been less extensively studied in other synucleinopathies. DLB shares many clinical and pathological features with PD, which has led researchers to group them under the broader category of Lewy body dementias [61] However, other studies argue that certain clinical and pathological characteristics, such as the presence of co-pathologies in specific brain regions, remain distinct enough to differentiate the two conditions [6].

Bentivegna et al. applied α-syn SAA to 871 antemortem CSF samples and 138 brain homogenates from an autopsy cohort [62]. These samples represented a range of neuropathological diagnoses, including DLB, but also Creutzfeldt-Jakob disease (the predominant pathology in the series), AD, MSA, and others [62]. Interestingly, when DLB pathology had extended to the limbic structures and neocortex (corresponding to Braak stages 4–6), α-syn RT-QuIC demonstrated 100% sensitivity [14,62,63]. In contrast, sensitivity was considerably lower in earlier stages—37.5% for Braak stages 1–2 and 73.3% for stage 3 [14,62,63]. Cases with focal DLB pathology limited to the amygdala also showed reduced sensitivity (50.0%) [62]. Notably, 8% of confirmed Creutzfeldt-Jakob disease cases exhibited incidental α-syn SAA positivity [62]. Furthermore, TTT appeared to correlate positively with the α-synuclein concentration in brain homogenates, suggesting its potential utility for estimating α-synuclein burden quantitatively [62]. Similarly, Samudra et al. tested antemortem CSF samples from 56 autopsy-confirmed cases using α-syn SAA [64]. The assay demonstrated high sensitivity (100%) and specificity (96.3%) in advanced, diffuse stages of the disease, where pathology had already spread to the cortex [64]. However, sensitivity was markedly lower when pathology was confined predominantly to the amygdala (42.8%) or brainstem (16.7%) [64]. Furthermore, α-syn SAA positivity was more frequently observed when the pathology extended beyond these localized regions [64].

Coughlin et al. applied α-syn SAA to the CSF of 191 patients with clinically possible or probable DLB, along with 99 healthy controls [4,65]. The assay demonstrated modest sensitivity (71.7%) and high specificity (96.0%) [65]. Within the patient group, α-syn SAA positivity was associated with more advanced clinical features, including lower MoCA scores, higher MDS-UPDRS III scores, and lower University of Pennsylvania Smell Identification Test scores [65]. These findings suggest that α-syn SAA positivity correlates with more clinically advanced disease. Interestingly, hyposmia—as measured by University of Pennsylvania Smell Identification Test—yielded the highest positive predictive value for α-syn SAA positivity [65]. Notably, of the 82 patients who underwent a follow-up CSF α-syn SAA, all but one showed the same result as their initial test [65].

The association between genomic DNA alterations and α-syn SAA positivity has also been investigated. Koss et al. successfully detected pathological α-syn within the neuronal nuclei of the temporal cortex in autopsy-confirmed DLB cases using α-syn SAA [66]. Notably, the presence of nuclear α-syn coincided with specific DNA alterations, particularly DNA double-strand break repair within the same cells [66]. Other cellular alterations may also be associated with DLB, and α-syn SAA appears to correlate with some of these changes. Specifically, Kon et al. conducted transcriptome analysis of the substantia nigra in autopsy-confirmed DLB cases [67]. Based on α-syn SAA positivity—measured by the number of positive replicates—they classified patients into high- and low-seeder subgroups. Notably, in the high-seeders group, the most significantly upregulated or downregulated genes were involved in membrane transport, lipid metabolism, and the ubiquitin–proteasome system [67].

### 2.4. Studies Investigating Multiple Synucleinopathies

#### 2.4.1. Cerebrospinal Fluid

As in studies focusing exclusively on PD, CSF remains the most extensively studied biological fluid for α-synuclein SAA in research involving multiple synucleinopathies. Table 3 summarizes the main findings from studies that analyzed CSF samples from cohorts including patients with more than one type of synucleinopathy.

Groveman et al. initially tested α-syn SAA in the CSF of autopsy-confirmed cases of PD and DLB, comparing them to autopsy-confirmed cases with non-synucleinopathic etiologies (including AD) [68]. The assay demonstrated high sensitivity and specificity for detecting Lewy body diseases [68]. Subsequently, Arnold et al. utilized α-syn SAA in the CSF of 119 autopsy-confirmed cases, the majority of which had either AD pathology (43 patients) or AD with α-syn co-pathology (59 patients) [69]. α-syn SAA was also applied in frontal cortex and amygdala homogenates. As previously seen in the studies of Samudra et al. and Bentivegna et al., α-syn SAA showed high sensitivity and specificity in cases with neocortical or limbic α-syn pathology, but sensitivity was disappointingly low in amygdala-predominant cases [62,64,69]. Higher positivity was also seen in brain homogenates from limbic/neocortical cases [69].

To evaluate the reproducibility of CSF α-syn SAA, Bräuer et al. collected samples from patients clinically diagnosed with PD or DLB [70]. Each sample was collected twice—once processed in one laboratory, and again in a second, separate laboratory. The qualitative results and kinetic parameters were consistent between the two laboratories, apart from a single patient [70]. Additionally, the investigators observed a negative correlation between α-syn SAA kinetic parameters and the degree of cognitive impairment [70].

The differential diagnosis between PD and atypical parkinsonian disorders, including MSA, can be particularly challenging. To address this, Fernandes Gomes et al. evaluated the diagnostic utility of CSF α-syn SAA in patients clinically diagnosed with PD, MSA, progressive supranuclear palsy (PSP), corticobasal degeneration (CBD), and healthy controls [71]. The assay demonstrated high sensitivity and specificity for both PD and MSA [71]. However, its ability to distinguish between PD and MSA was limited, with notably poor sensitivity [71]. Furthermore, the specificity for PD was reduced when compared with PSP, CBD, and healthy controls [71].

Differentiating PD from MSA using CSF α-syn SAA remains challenging. Wiseman et al. analyzed CSF samples and brain homogenates (from the medulla, substantia nigra, cerebellum, hippocampus, and middle temporal gyrus) obtained from autopsy cases of patients with PD, MSA, and healthy controls [72]. Notably, CSF samples were available from only eight individuals, all diagnosed with PD. The α-syn SAA measured in brain tissue successfully distinguished MSA from PD, based on higher kinetic parameters observed in MSA samples—specifically, protein aggregation rate (PAR), core protofilament size, gradient of amplification (GA), and Fmax [72]. Of these, only PAR was significantly elevated in the CSF of PD patients compared to corresponding brain homogenates [72]. Similarly, Ma et al. investigated the presence of α-syn SAA in CSF samples and brain tissues from autopsy-confirmed or clinically diagnosed cases of PD, MSA, DLB, iRBD, non-synucleinopathic neurodegenerative diseases (including CBD, PSP, argyrophilic grain disease, among others), as well as healthy controls, across eight different cohorts [73]. They identified three distinct categories of α-syn SAA positivity and fluorescence patterns: high in Lewy body diseases, intermediate in cases with glial cytoplasmic inclusions, PSP, and CBD, and below-threshold in negative cases [73]. Interestingly, Al-Lahham et al. analyzed CSF and brain samples from patients with DLB and MSA and identified distinct α-synuclein aggregate morphologies between the two diseases, reflected in their differing kinetic parameters.

Multiple pathologies frequently co-exist in neurodegenerative diseases [76] especially in Lewy body disease [77]. To investigate this, Plastini et al. calculated α-synuclein SAA in CSF samples from patients diagnosed with either DLB, PD, PD dementia—PDD or AD, following them longitudinally over a ten-year period [75]. The assay demonstrated high sensitivity, specificity, and overall accuracy for distinguishing Lewy body disease from non-Lewy body pathologies [75]. Interestingly, α-syn SAA was also positive in 12% of AD cases, as determined by disease-specific biomarkers, suggesting the presence of co-pathology in a subset of these patients [75].

#### 2.4.2. Other Biological Tissues

Table 4 summarizes key findings from studies that analyzed non-CSF samples from cohorts including patients with more than one type of synucleinopathy.

Some studies have examined α-syn SAA exclusively in brain tissue, without including CSF samples. Yoshinaga et al. analyzed brain tissue from the insular gyrus of patients with DLB, MSA, and healthy controls [78]. They concluded that the structural characteristics of the amplified α-synuclein fibrils could contribute to the study of α-synuclein aggregates in neurodegenerative diseases. However, these features did not allow for differentiation between DLB and MSA cases [78]. Subsequently, Martinez-Valbuena et al. applied α-synuclein SAA to autopsy-confirmed cases of PD, MSA, PSP, and healthy controls [79]. By using specific buffer conditions, they were able to classify MSA patients into three distinct groups based on seeding activity: high, intermediate, and low seeders [79]. Moreover, Kim et al. evaluated α-synuclein SAA in formaldehyde-fixed, paraffin-embedded samples from the substantia nigra, cerebellum, and temporal cortex of autopsy-confirmed DLB and MSA cases [80]. Using a streamlined protein extraction protocol, they were able to detect disease-specific seeding activity [80]. Similarly, Fenyi et al. investigated α-syn SAA in autopsy-confirmed cases of PD, DLB, and healthy controls [81]. They analyzed both brain homogenates and peripheral tissue samples from the gastric cardia, including the adventitia, muscle layers, submucosa, and Auerbach’s plexus [81]. Their findings revealed that the TTT was significantly shorter in patients over 80 years of age [81].

Regarding the periphery, the most extensively investigated tissue is in the oral cavity. Zheng et al. applied α-syn SAA to specimens from the oral mucosa of patients with PD, MSA, iRBD, and healthy controls [82]. High specificity but low sensitivity was achieved for PD patients against controls, while positivity for MSA patients was also low [82]. On the other hand, Luan et al. utilized saliva samples from patients with PD, MSA, and healthy controls [83]. While the assay demonstrated high specificity for PD compared to controls, sensitivity was low for both diseases [83]. Interestingly, although the characteristics of α-syn fibrils did not differ significantly between PD and MSA, the TTT was significantly shorter in the PD subgroup compared to MSA [83]. Subsequently, the same research group conducted α-syn SAA on saliva samples from patients with PD, MSA, essential tremor, and healthy controls, achieving sensitivity and specificity comparable to their previous study [84].

## 3. Discussion

Most studies investigating the diagnostic utility of α-syn SAA in synucleinopathies have focused primarily on patients with PD. CSF was the most tested sample type. Participants represented a broad range of disease stages: some studies included individuals at risk for developing PD, patients with denovo or early-stage PD, while others involved individuals with moderate to advanced disease. As a result, one could argue that these studies collectively offer insights across the full clinical spectrum of PD.

CSF α-syn SAA have demonstrated high sensitivity and specificity in most studies, with specificity typically slightly exceeding sensitivity when compared to control groups. However, these strong diagnostic performance metrics are primarily observed in patients with established PD. In contrast, the diagnostic utility of α-syn SAA in prodromal or at-risk populations is generally modest or low [23,33,35,36]. Notably, it is not uncommon for individuals who test positive for α-syn SAA but are only at risk to remain free of clinically manifest synucleinopathy, even after several years of longitudinal follow-up [36]. This also applies to individuals presenting with multiple premotor symptoms, such as three or more, where phenoconversion remains relatively infrequent [35]. Nonetheless, combining α-syn SAA with additional diagnostic methods, such as DAT-SPECT, objective assessments of prodromal symptom severity (e.g., hyposmia), and specific characteristics of the α-syn SAA assay (including kinetic parameters and the number of positive replicates), may enhance the identification of individuals at higher risk of phenoconversion [26,30,36]. Despite this, current evidence does not support the use of α-syn SAA alone in prodromal stages of synucleinopathies for reliable clinical counseling regarding the risk of developing clinically overt disease. A potential future application lies in genetically defined at-risk populations. In these groups, α-syn SAA positivity appears to vary significantly depending on the specific mutation, suggesting a more nuanced role in genetic subtypes [28,31].

Furthermore, while the diagnostic accuracy of CSF α-syn SAA appears satisfactory in established PD, this is not consistently observed with other biological sample types. Although specificity remains relatively high, sensitivity is often modest or low in tissues such as the gastrointestinal tract and saliva [52,53,54,56], with skin offering comparatively better sensitivity [55,57].

This outcome is somewhat unexpected given the Braak staging hypothesis, which proposes that the pathological process begins in peripheral tissues and subsequently spreads to the brain [14,63]. Based on this model, one might anticipate that peripheral tissues—where pathology is thought to emerge years before motor symptoms appear—would exhibit higher α-syn SAA positivity rates than CSF. However, this does not seem to be the case.

Blood-based assays have demonstrated high sensitivity and specificity, as they capture α-syn derived directly from the CNS, essentially functioning as CNS “droplets” outside the CNS [49,85]. Nevertheless, this approach requires highly specialized techniques, and unlike CSF α-syn SAA, the reproducibility of blood-based SAA using NEs has yet to be validated across laboratories [40,49,70,85]. Therefore, improving the sensitivity of α-syn SAA in specimens other than CSF, along with ensuring reproducibility and standardization of these procedures, is essential before such methods can be implemented in routine clinical practice.

Nevertheless, CSF α-syn SAA appears to be a reliable method that may aid in the diagnosis of clinically established synucleinopathies. However, other diagnostic biomarkers, such as DAT-SPECT in PD and FDG-PET in DLB, are already approved and routinely used in clinical practice to support or exclude a diagnosis of synucleinopathy [4,5]. It remains unproven whether α-syn SAA offers superior diagnostic utility compared to these established techniques, aside from limited data suggesting potential advantages over DAT-SPECT in PD [24,40,42]. Well-designed, head-to-head studies comparing the diagnostic performance of CSF α-syn SAA with other existing biomarkers are needed to clarify its clinical value.

The diagnostic landscape becomes even more unclear when considering synucleinopathies beyond PD. Although α-syn SAA have demonstrated exceptionally high sensitivity and specificity in advanced-stage DLB cases, their performance in earlier stages has been disappointing [62,64]. Even when brain homogenates from autopsy-confirmed cases were analyzed, the sensitivity of the method remained low if the pathology was confined to the initial sites of disease manifestation, such as the amygdala or brainstem [62,64]. This limitation presents a significant challenge for diagnostic utility, as early and accurate diagnosis is crucial for initiating timely therapeutic interventions and for appropriately classifying patients in future clinical trials. In MSA, data mainly come from studies including MSA, PD, and/or DLB cases rather than MSA alone. Nonetheless, CSF α-syn SAA has shown good specificity and moderate sensitivity for MSA diagnosis [71,73]. As in PD and DLB, diagnostic accuracy in other tissue types remains limited [83,84].

Studies that included samples from multiple synucleinopathies have generally failed to distinguish between PD, DLB, and MSA when relying solely on α-syn SAA positivity—a predictable outcome, as SAA is a qualitative method and all three conditions typically yield high positivity rates [69,71,73]. However, when the number of positive replicates or specific kinetic parameters are analyzed or when SAA is combined with other protein-based techniques, several studies have successfully differentiated between synucleinopathies [70,72,73,74]. Kinetic parameters may also be associated with the severity of several motor and non-motor symptoms, suggesting their utility in monitoring disease progression [42,47,70]. These approaches, however, require more advanced and complex methodologies that are not yet standardized across laboratories, raising concerns about reproducibility [21,70].

The observation that SAA sensitivity is often lower in peripheral tissues such as the gastrointestinal tract or skin, compared to cerebrospinal fluid, appears to contrast with Braak’s hypothesis suggesting a peripheral origin of α-synuclein pathology [14]. Several factors may account for this discrepancy. Technical limitations in sample collection, processing, and assay performance in peripheral matrices may reduce sensitivity. Additionally, temporal dynamics of α-synuclein propagation could mean that peripheral seeding activity declines by the time central pathology becomes detectable. Biological differences in aggregate conformation, concentration, or compartmentalization between peripheral and central tissues may also impact detectability [86,87,88,89,90,91,92]. Further research is needed to clarify these mechanisms.

Furthermore, when synucleinopathies are compared to other non-synucleinopathic neurodegenerative diseases, some studies have demonstrated promising diagnostic accuracy [57,68,69,73]. However, it is important to acknowledge that several other studies have reported positive α-syn SAA results in diseases outside the synucleinopathy spectrum. Beyond AD—where α-syn is frequently observed as a co-pathology alongside amyloid and may even contribute to disease progression—recent studies have detected positive α-syn SAA findings in a range of neurodegenerative disorders with distinct primary pathologies, including CBS, PSP, Creutzfeldt-Jakob disease, and even amyotrophic lateral sclerosis [62,93,94,95,96,97,98,99,100,101]. These findings do not necessarily suggest a lack of specificity in the α-syn SAA method. In fact, the studies included in the present review generally support the high specificity of α-syn SAA when distinguishing synucleinopathies from healthy controls without neurodegenerative disease [39,42,53,54,68,69]. What may be occurring instead is the presence of α-synuclein co-pathology in diseases beyond AD, reflecting a broader phenomenon of mixed pathologies. Indeed, some researchers propose that co-pathology is the rule rather than the exception in neurodegenerative disorders [76,102,103]. Therefore, in cases where diagnostic uncertainty persists despite α-syn SAA results, it may be prudent to improve diagnostic discrimination by integrating α-syn SAA with additional biomarkers of neurodegeneration, such as phosphorylated tau, amyloid-beta, and neurofilament light chain [104,105].

Additionally, while co-pathology remains a plausible explanation for SAA positivity in disorders such as PSP, CBD, Creutzfeldt-Jakob disease or amyotrophic lateral sclerosis, alternative mechanisms may also contribute and merit discussion. One possibility is the presence of subclinical or early-stage synucleinopathy that has not yet manifested with overt clinical or pathological hallmarks [106]. Additionally, shared or convergent molecular pathways, such as neuroinflammation, mitochondrial dysfunction, or impaired proteostasis, may create an environment conducive to cross-seeding or the misidentification of pathological proteins in SAA assays [107]. Cross-reactivity of the assay or the presence of structurally similar aggregates, especially in rapidly progressive or atypical neurodegenerative diseases like CJD, could also contribute to false positives [108]. Furthermore, recent studies have demonstrated α-synuclein aggregation in a subset of ALS and FTD patients, suggesting possible overlap or convergence of proteinopathies [103,109]. Considering these alternative explanations helps refine SAA result interpretation and underscores the need for complementary diagnostic approaches. Further research is warranted to clarify these complex pathological overlaps and refine biomarker-based diagnostics.

Several practical challenges must be addressed before SAA can be adopted into routine diagnostic workflows. These include the need for protocol standardization and inter-laboratory validation to ensure reproducibility and comparability of results across centers. Additionally, the assays currently rely primarily on CSF, which requires an invasive lumbar puncture and may limit patient acceptance. Diagnostic accuracy also varies in prodromal and peripheral tissue samples, and the ability to distinguish between different synucleinopathy subtypes remains limited. Furthermore, interpreting SAA results in the context of co-pathologies adds complexity, and the cost and technical demands of the assay may restrict widespread accessibility. Addressing these issues will be essential to fully realize the clinical utility of SAA in the diagnosis and management of synucleinopathies.

Interestingly, the findings of our narrative review align with recent reviews and meta-analyses on SAA in PD, which report that the highest diagnostic accuracy is achieved using CSF. Among peripheral tissues, blood-derived extracellular vesicles and skin biopsies have shown the most promising results, while other specimens, such as saliva and gastrointestinal tract biopsies, generally demonstrate lower reliability [110,111,112]. Regarding other synucleinopathies, CSF—consistent with our findings—appears to be a promising diagnostic tool for both PD and DLB. However, results remain more controversial for MSA, peripheral tissue samples, and prodromal forms of synucleinopathies, where sensitivity and specificity tend to be lower and more variable across studies [113,114,115,116].

Of course, our review has several limitations. First, we restricted our literature search to studies indexed in PubMed. As a result, relevant studies available exclusively in other scientific databases may have been inadvertently excluded. Secondly, the study screening and selection process was conducted by a single researcher, which may introduce a risk of selection bias. Thirdly, the studies included in this narrative review exhibit substantial heterogeneity in SAA protocols, sample characteristics, and patient populations. This variability suggests that a formal assessment of study quality and risk of bias would be more appropriate within the scope of a systematic review.

## 4. Materials and Methods

### 4.1. Search Strategy

The PubMed database was searched from inception to 9 June 2025, using the exact search algorithm: “seed amplification assay synuclein.” Studies were included if they met the following criteria: (a) original research articles; (b) full text available in English; (c) human studies; (d) reporting the number of patients from each synucleinopathy and the tissue source of their samples; and (e) investigations assessing the diagnostic utility of α-synuclein SAA in patients with, or at risk of developing, synucleinopathies (PD, DLB, or MSA). Case reports, brief communications, opinion letters, letters to the editor, systematic reviews, narrative reviews, meta-analyses and studies enrolling only patients with other neurodegenerative diseases, apart from synucleinopathies or studies irrelevant to our research purpose were excluded from the review.

### 4.2. Study Selection and Categorization

**Figure 1:**
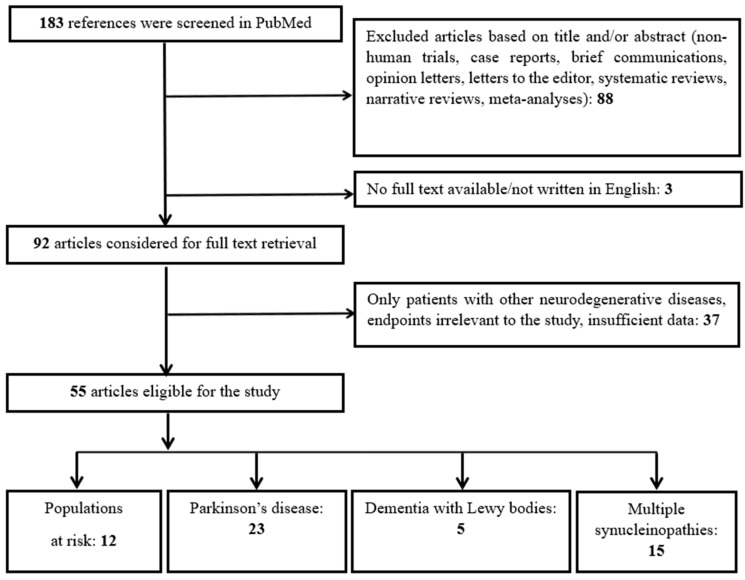
Flow-chart of study selection and categorization.

As depicted in Figure 1, our algorithm initially generated 183 results. Three clinical trials were subsequently excluded due to the absence of a full text or abstract, or because they were not published in English. Furthermore, 88 articles were excluded for not meeting the criteria of original research papers. Of the remaining 92 articles, 37 additional studies were deemed irrelevant or did not involve patients with, or at risk for, synucleinopathy or provided insufficient data.

Consequently, 55 clinical studies comprised the final literature review. For analysis, these studies were categorized by their specific scope into four groups: (a) populations at risk for synucleinopathy (12 studies); (b) studies with PD patients (23 studies); (c) studies with DLB (5 studies); and (d) studies involving patient groups with multiple synucleinopathies (15 studies).

## 5. Conclusions

In conclusion, α-syn SAA show significant diagnostic potential in synucleinopathies, with the most promising application in the detection of early PD and CSF emerging as the most reliable specimen type. Additional applications may include monitoring disease progression, although this requires further validation. Future studies should explore the utility of α-syn SAA in alternative biological specimens, assess its performance across various synucleinopathies and other neurodegenerative diseases, and determine its comparative diagnostic value. Enhancing the discriminative power of the assay may be possible by analyzing kinetic parameters or combining α-syn SAA with other biomarkers. However, implementing these strategies necessitates advanced methodological expertise and robust validation to ensure reproducibility and clinical reliability. Given the inherent heterogeneity in SAA protocols, sample characteristics, and patient populations across the included studies, a formal assessment of study quality and risk of bias—ideally as part of a subsequent systematic review—would help to further validate and standardize the conclusions drawn from the current research.

Furthermore, challenges such as the need for protocol standardization, inter-laboratory validation, high cost, reliance on invasive CSF sampling, limited accuracy in early or peripheral samples, and difficulty distinguishing subtypes or interpreting co-pathologies must be addressed. Overcoming these hurdles is crucial for successful clinical integration.

Looking ahead, several research priorities are essential to advance the clinical utility of SAA. These include multi-center standardization and validation studies to ensure assay reliability, and longitudinal investigations in prodromal cohorts to assess early diagnostic value. Head-to-head comparisons with established biomarkers (e.g., DAT-SPECT, FDG-PET, MRI) will clarify SAA’s relative strengths. Developing robust blood-based assays could improve accessibility. Further, understanding the influence of genetic variants (e.g., *GBA1*, *LRRK2*) on SAA performance, exploring its use in disease stratification and progression monitoring, and delineating peripheral versus central seed dynamics will be critical future directions.

## Figures and Tables

**Table 1:** Overview of studies analyzing CSF ^1^ in PD ^2^ using α-syn ^3^ SAA ^4^.

Study	PD Patient Numbers	Additional Parameters Tested	Main Findings	Study Notes
Shahnawaz et al., 2017 [37]	76	Fmax ^5^, TTT ^6^, T50 ^7^, AUC ^8^	-88.5% sensitivity and 96.9% specificity for PD patients compared to other neurological disorders -negative correlation between T50 and H&Y ^9^ for the PD group	-relatively large sample size -cohort study -absence of autopsy-confirmed cases
Bhumkar et al., 2021 [38]	8	-	-75% positivity for PD patients compared to HC	-small sample size -absence of autopsy-confirmed cases
Orrù et al., 2021 [39]	108	Fmax, TTT, T50, AUC	97% positivity for PD patients and AUC 0.95 compared to HC ^10^	-relatively large sample size -retrospective study -absence of autopsy-confirmed cases
Russo et al., 2021 [40]	30	Fmax, TTT, AUC	86–96% sensitivity and 93–100% specificity for PD compared to parkinsonian patients with normal DAT-SPECT ^11^ and HC	-small sample size -cohort study -absence of autopsy-confirmed cases
Oftedal et al., 2023 [41]	121	-	82.6% sensitivity and 88.2% specificity for PD compared to HC	-relatively large sample size -cohort study -absence of autopsy-confirmed cases
Majbour et al., 2022 [42]	111	Fmax, T50, AUC, α-syn oligomer-specific ELISA ^12^	-SAA product quantification with oligomeric ELISA 100% sensitivity and specificity for PD patients against HC -positive correlation between α-syn oligomer levels and H&Y and MDS-UPDRS III ^13^ for PD patients	-relatively large sample size for PD patients -cohort study -29 autopsy-confirmed cases
Middleton et al., 2023 [43]	96	-	-83.9% diagnostic accuracy for PD patients compared to HC using only clinical diagnosis -93.6% diagnostic accuracy for PD patients compared to HC combining clinical diagnosis with confirmatory DAT- SPECT	-relatively large sample size -retrospective cohort study -absence autopsy-confirmed cases
Bellomo et al., 2023 [44]	4	Fmax, T50, Fmin ^14^	-strong inhibition of α-syn aggregation by apolipoproteins ApoA1 and ApoE compared to HC -observation of α-syn–apolipoprotein complexes -correlations between ApoA1 and ApoE levels and SAA kinetic parameters compared to HC	-small sample size -absence of autopsy-confirmed cases
Gaetani et al., 2025 [45]	47	-	-no difference in SNAP25 ^15^ levels in serum and CSF, and VAMP2 ^16^ levels in CSF, in AD patients with α-syn SAA positivity compared to AD patients with α-syn SAA negativity -higher SNAP25 levels in CSF and serum in PD patients positive for AD ^17^ pathology compared to PD patients negative for AD pathology	-relatively large sample size -retrospective cohort study -absence of autopsy-confirmed cases
Eijsvogel et al., 2024 [46]	20	Fmax	reduction in CSF α-syn seeds in PD treatment group compared to PD control group	-small sample size -randomized controlled trial -absence of autopsy-confirmed cases
Grillo et al., 2025 [47]	547	Fmax, TTT, T50, AUC, RFU ^18^, RFU/hours	-77% positivity for *LRRK2* ^19^ patients, 92.3% for *GBA1* ^20^ PD, 93.8% for sporadic PD -longer T50 and TTT, smaller AUC for *LRRK2* compared to *GBA1* and sporadic PD -shorter T50 and larger AUC for sporadic PD patients with dysautonomia compared to those without	-relatively large sample size -cohort study -absence of autopsy-confirmed cases -absence of HC
Bräuer et al., 2025 [48]	34	Fmax, TTT, AUC, RFU, time to 2 positive replicates, cultured cell transfection with SAA products	two types of α-syn aggregates, based on kinetic profiles and cell seeding capacities for PD patients	-small sample size -cross sectional study -absence of autopsy-confirmed cases

^1^ CSF: cerebrospinal fluid, ^2^ PD: Parkinson’s disease, ^3^ α-syn: α-synuclein, ^4^ SAA: seed amplification assays, ^5^ Fmax: maximum fluorescence, ^6^ TTT: time-to-threshold, ^7^ T50: time-to-50% maximum fluorescence, ^8^ AUC: area under the curve, ^9^ H&Y: Hoehn and Yahr, ^10^ HC: healthy controls, ^11^ DAT-SPECT: dopamine transporter single-photon emission computed tomography, ^12^ ELISA: Enzyme-linked Immunosorbent Assay, ^13^ MDS-UPDRS III: Movement Disorders Society Unified Parkinson’s Disease Rating Scale Part III, ^14^ Fmin: minimum fluorescence, ^15^ SNAP25: synaptosomal-associated protein 25 kDa, ^16^ VAMP2: vesicle-associated membrane protein 2, ^17^ AD: Alzheimer’s disease, ^18^ RFU: relative fluorescence unit, ^19^ *LRRK2*: Leucine-rich repeat kinase 2, ^20^ *GBA1*: beta glucocerebrosidase.

**Table 2:** Overview of studies analyzing samples other than CSF ^1^ in PD ^2^ using α-syn ^3^ SAA ^4^.

Study	Tissue Examined	PD Patient Numbers	Additional Parameters Tested	Main Findings	Study Notes
Schaeffer et al., 2024 [49]	Blood	80	Fmax ^5^, TTT ^6^, T50 ^7^, AUC ^8^, F60 ^9^	-98.8% sensitivity for PD patients compared to HC ^10^ -lower F60 with longer disease duration compared to shorter disease duration for PD patients	-relatively large sample size for the PD group -cross-sectional study -absence of autopsy-confirmed cases
Kluge et al., 2024 [25]	Blood	22	-	-61.5% of *PRKN* mutation carriers positive for α-syn SAA compared to HC	-small sample size -cross-sectional study -absence of autopsy-confirmed cases
Daida et al., 2024 [50]	Blood	875	-	-greater SAA activity for *V15A*-derived α-syn fibrils compared to wild-type α-syn	-large sample size, but only two families with the *V15A* variant -cross-sectional study -absence of autopsy-confirmed cases
Wang et al., 2024 [51]	Blood, saliva	187 (82 blood only, 48 blood and saliva, 57 saliva only)	-	-80.5% sensitivity, 90.5% specificity, 0.90 accuracy for PD patients compared to HC in the serum -74.7% sensitivity, 97.9% specificity, 0.90 accuracy for PD patients compared to HC in the saliva -95.8% sensitivity, 96.1% specificity, 0.98 accuracy for PD patients compared to HC with serum and saliva combined	-relatively large sample size for the PD group -prospective study -absence of autopsy-confirmed cases
Chahine et al., 2023 [52]	Saliva, CSF	59	Fmax, T50, AUC	-92.6% sensitivity and 90.5% specificity for PD patients compared to HC in the CSF -73.2% sensitivity and 78.6% specificity for PD patients compared to HC in the saliva -65.8% positivity of PD patients compared to HC for α-syn SAA in both CSF and saliva	-relatively large sample size for the PD group -cohort study -absence of autopsy-confirmed cases
Vascellari et al., 2023 [53]	Duodenum biopsies	23	SD50 ^11^	95.7% sensitivity and 100% specificity for PD patients compared to HC in duodenum biopsies	-small sample size -all PD patients in advanced stage -cross-sectional study -absence of autopsy-confirmed cases
Fenyi et al., 2019 [54]	Antrum, sigmoid colon or rectum biopsies	18	TTT	-55.6% sensitivity and 100% specificity for PD patients compared to HC in gastrointestinal biopsies -70% of positive results in sigmoid colon biopsies for patients compared to HC	-small sample size -cross-sectional study -absence of autopsy-confirmed cases
Emmi et al., 2025 [55]	Gastric, duodenum, and skin biopsies	22	Fmax, TTT	-87.7% accuracy in skin, 67.4% in duodenum, 80.0% in gastric biopsies for PD patients compared to HC -81.8% sensitivity in skin, 88.9% in gastric, 58.8% in duodenal biopsies in advanced PD patients compared to HC	-small sample size -cohort study -absence of autopsy-confirmed cases
Shin et al., 2022 [56]	Upper and lower gastrointestinal tract	20	RFU	10% positivity of gastric biopsies from PD patients compared to none in HC	-small sample size -retrospective study -only 6 cases were autopsy-confirmed
Mao et al., 2025 [57]	Skin, brain	214 (skin), 14 (brain)	TTT, quiescent SAA	90.2% sensitivity and 91.4% specificity in the skin samples for PD patients compared to non-PD cases and HC	-relatively large sample size for PD patients -retrospective study -only 20 cases were autopsy-confirmed

^1^ CSF: cerebrospinal fluid, ^2^ PD: Parkinson’s disease, ^3^ α-syn: α-synuclein, ^4^ SAA: seed amplification assays, ^5^ Fmax: maximum fluorescence, ^6^ TTT: time-to-threshold, ^7^ T50: time-to-50% maximum fluorescence, ^8^ AUC: area under the curve, ^9^ F60: fluorescence intensity at the endpoint of the amplification assay, ^10^ HC: healthy controls, ^11^ SD50: tissue concentrations of seeding units giving 50% positive replicate reactions.

**Table 3:** Overview of studies analyzing CSF ^1^ in multiple synucleinopathies using α-syn ^2^ SAA ^3^.

	Type of Synucleinopathy and Patient Numbers (n)	Additional Parameters Tested	Main Findings	Study Notes
Groveman et al., 2018 [68]	PD ^4^ (12), DLB ^5^ (17)	-	93% sensitivity, 100% specificity for PD and DLB patients compared to non-synucleinopathic neurodegenerative diseases	-small sample size for all study groups -prospective study -pathological confirmation in some cases (specific pathology undefined)
Arnold et al., 2022 [69]	PD (4), DLB (9)	Fmax ^6^, TTT ^7^, T50 ^8^, RFU ^9^	-97.8% sensitivity, 98.1% specificity for limbic and/or neocortical synuclein pathology compared to non-synucleinopathic pathologies -14.3% sensitivity for amygdale-predominant α-syn pathology compared to non-synucleinopathic pathologies	-small sample size for PD and DLB study groups -retrospective study -all cases where pathologically confirmed
Bräuer et al., 2023 [70]	PD (28), DLB (47)	Fmax, TTT, RFU, AUC ^10^, TT1 ^11^, TT2 ^12^	-consistent kinetic parameters between the two laboratories in 98% of patients -negative correlation between TTT and TT2 and MoCA ^13^ score for both PD and DLB	-small sample size for PD study group, relatively large for DLB -cross-sectional study -absence of pathological confirmation
Fernandes Gomes et al., 2023 [71]	PD (55), MSA ^14^ (27)	Fmax, Fmin ^15^, RFU	-100% sensitivity for PD compared to HC ^16^, 92.6% positivity for MSA compared to HC -7% specificity for PD compared to MSA, 70.8% to HC, 71% to CBD ^17^, and 75% to PSP ^18^ compared to HC	-small sample size for all study groups -retrospective study -absence of pathological confirmation
Wiseman et al., 2025 [72]	PD (10), MSA (10)	Fmax, Fmin, Fmaxr ^19^ TTT, 1/TTT, AUC, PAR ^20^, GA ^21^, core protofilament size	-higher Fmax, PAR, GA, and core protofilament size in brain tissues of MSA compared to PD patients -higher PAR in PD CSF samples compared to brain tissue	-small sample size for all study groups -retrospective study -all cases where pathologically confirmed
Ma et al., 2024 [73]	PD (109), DLB (15), MSA (111)	Fmax, RFU	-three patterns of α-syn SAA fluorescence: high in Lewy body diseases, intermediate in MSA, PSP, and CBD, below-threshold in negative cases -95% sensitivity and specificity for PD compared to negative cases -84–91% sensitivity, 68–87% specificity for MSA among different cohorts compared to negative cases	-relatively large sample size for PD and MSA, small for DLB -retrospective study -all cases where pathologically confirmed
Al-Lahham et al., 2025 [74]	DLB (3), MSA (3)	Fmax	-distinct α-syn SAA kinetics between DLB and MSA	-small sample size for all study groups -retrospective study -all cases where pathologically confirmed
Plastini et al., 2024 [75]	PD, PDD ^22^ and DLB (150)	RFU	-89.3% sensitivity, 93.3% specificity, and 91.3% accuracy for PD, PDD, and DLB patients compared to AD ^23^ -12% positivity in AD cases	-relatively large sample size, but synucleinopathies were not discriminated -retrospective study -only two cases were autopsy-confirmed

^1^ CSF: cerebrospinal fluid, ^2^ α-syn: α-synuclein, ^3^ SAA: seed amplification assays, ^4^ PD: Parkinson’s disease, ^5^ DLB: dementia with Lewy bodies, ^6^ Fmax: maximum fluorescence, ^7^ TTT: time-to-threshold, ^8^ T50: time-to-50% maximum fluorescence, ^9^ RFU relative fluorescence units, ^10^ AUC: fluorescent area under the curve, ^11^ TT1: time to 1 positive replicate, ^12^ TT2: time to 2 positive replicates, ^13^ MoCA: Montreal Cognitive Assessment, ^14^ MSA: multiple system atrophy, ^15^ Fmin: minimum fluorescence, ^16^ HC: healthy controls, ^17^ CBD: corticobasal degeneration, ^18^ PSP: progressive supranuclear palsy, ^19^ Fmaxr: maximum relative fluorescence, ^20^ PAR: protein aggregation rate, ^21^ GA: gradient of amplification, ^22^ PDD: Parkinson’s disease dementia, ^23^ AD: Alzheimer’s disease.

**Table 4:** Overview of studies analyzing samples other than CSF ^1^ in multiple synucleinopathies using α-syn ^2^ SAA ^3^.

	Type of Synucleinopathy and Patient Numbers (n)	Tissue Examined	Additional Parameters Tested	Main Findings	Study Notes
Yoshinaga et al., 2020 [78]	DLB ^4^ (5), MSA ^5^ (5)	Brain	-	α-syn aggregates structural properties could differentiate DLB from MSA	-small sample size for all study groups -retrospective study -all cases where pathologically confirmed
Martinez-Valbuena et al., 2022 [79]	PD ^6^ (15), MSA (15)	Brain	-	MSA categorization to high, intermediate, and low seeders compared to PD, PSP ^7^, and HC ^8^	-small sample size for all study groups -retrospective study -all cases where pathologically confirmed
Kim et al., 2023 [80]	DLB (6), MSA (2)	Brain	-	disease-specific seeding activity with a streamlined protein extraction protocol in DLB and MSA compared to HC	-small sample size for all study groups -retrospective study -all cases where pathologically confirmed
Fenyi et al., 2021 [81]	PD (12), DLB (8)	Brain, enteric nervous system	TTT ^9^	Shorter TTT in patients over 80 years of age compared to other age groups	--small sample size for all study groups -retrospective study -all cases where pathologically confirmed
Zheng et al., 2023 [82]	PD (107), MSA (99)	Oral mucosa	-	-67.3% sensitivity, 90.3% specificity for PD patients compared to HC -53.5% positivity in MSA patients compared to HC	-relatively large study groups -cross-sectional study -absence of pathologically confirmed cases
Luan et al., 2022 [83]	PD (75), MSA (18)	Saliva	TTT	-76.0% sensitivity, 94.4% specificity for PD compared to HC -61.1% sensitivity for MSA compared to HC -shorter TTT for PD compared to MSA	-small sample size for MSA -cross-sectional study -absence of pathologically confirmed cases
Luan et al., 2024 [84]	PD (101), MSA (32)	Saliva	TTT	70.30% sensitivity for PD compared to HC, 56.25% for MSA compared to HC, 92.45% specificity for HC	-relatively large study groups -cross-sectional study -absence of pathologically confirmed cases

^1^ CSF: cerebrospinal fluid, ^2^ α-syn: α-synuclein, ^3^ SAA: seed amplification assays, ^4^ DLB: dementia with Lewy bodies, ^5^ MSA: multiple system atrophy, ^6^ PD: Parkinson’s disease, ^7^ PSP: progressive supranuclear palsy, ^8^ HC: healthy controls, ^9^ TTT: time-to-threshold.

## Data Availability

No new data were created during the preparation of this manuscript.

## Abbreviations

The following abbreviations are used in this manuscript:

a-syn	alpha-synuclein
AD	Alzheimer’s disease
AUC	area under the curve
CBD	corticobasal degeneration
CNS	central nervous system
CSF	cerebrospinal fluid
DAT-SPECT	dopamine transporter single-photon emission computed tomography
DLB	dementia with Lewy bodies
ELISA	Enzyme-linked Immunosorbent Assay
FDG-PET	fluorodeoxyglucose—positron emission tomography
F60	fluorescence intensity at the endpoint of the amplification assay
Fmax	maximum fluorescence
Fmin	minimum fluorescence
GA	gradient of amplification
*GBA1*	beta-glucocerebrosidase
H&Y	Hoehn and Yahr scale
iRBD	idiopathic rapid eye movement sleep behavior disorder
*LRRK2*	leucine-rich repeat kinase 2
MDS-UPDRS III	Movement Disorders Society Unified Parkinson’s Disease Rating Scale Part III
MoCA	Montreal Cognitive Assessment
MSA	multiple system atrophy
NE	neuronal exosomes
PAR	protein aggregation rate
PD	Parkinson’s disease
PDD	Parkinson’s disease dementia
PSP	progressive supranuclear palsy
RT-QuIC	real-time quaking-induced conversion
SAA	seed amplification assay
SD50	tissue concentrations of seeding units giving 50% positive replicate reactions
SNAP25	synaptosomal-associated protein 25 kDa
*SNCA*	gene coding α-synuclein
T50	time-to-50% maximum fluorescence
TTT	time-to-threshold
VAMP2	vesicle-associated membrane protein 2
